# Sterile Corneal Ulcer and Sjögren’s Syndrome Associated with Long-Term Interferon alpha-2b Treatment in a Case of Multiple Myeloma

**Published:** 2006-09

**Authors:** Yin-Tsu Liu, Ko-Hua Chen, Wen-Ming Hsu

**Affiliations:** 1 *Department of Ophthalmology, Taipei Veterans General Hospital, Taiwan (ROC);*; 2 *National Yang-Ming University, Taiwan (ROC);*; 3 *Division of Medical Engineering, National Health Research Institutes, Taipei, 114, Taiwan (ROC)*

**Keywords:** corneal ulcer, interferon alpha-2b, dry eyes, sjögren’s syndrome, immunosuppressant

## Abstract

A 71-year-old Chinese male was referred for severe dry eyes who suffered from multiple corneal melting ulcers and underwent a long-term and high-dosage interferon alpha-2b (INFα-2b) treatment for multiple myeloma (MM). The patient was diagnosed to have MM 12 years ago, and since then INFα-2b (3-million units, intramuscularly, three times a week) was prescribed until the present. Two years ago, diffuse superficial punctuate epithelial defects, filaments and multiple melting ulcers in his cornea were noted. Sjögren’s syndrome was diagnosed after the salivary gland biopsy. In addition to preservative-free topical lubricants, bilateral permanent punctal occlusion and tarsorraphy were performed but in vain. Topical and systemic immunosuppressants including corticosteroids, cyclosporin and methotrexate improved his corneal condition successfully. The present case suggests a relationship between INFα-2b and Sjögren’s syndrome. Long-term use of INFα-2b may impair tear functions and complicate corneal melting ulcers. Immunosuppressants play a role in treatment of INFα-2b associated keratopathy.

## INTRODUCTION

Low-dose interferon alfa-2b (INFα-2b) is reported to improve tear function and xerostomia in patients of Sjögren’s syndrome(SS) (1, 2). However, a higher dosage of INFα-2b with a long-term administration is used in treatment of patients of hepatitis C (HC) and some blood diseases such as multiple myeloma (MM). In these patients, INFα-2b is reported to be related to some side effects including dry eyes and SS (3, 4).

Here, we present a case of MM who was treated with INFα-2b for nearly 12 years and in the recent 2 years, he was diagnosed with SS that compromised his tear films and corneas. He underwent the regular treatments for dry eyes including preservative-free lubricants, punctal occlusion, therapeutic contact lens, goggles, and tarsorraphy but in vain. Under the additional topical and systemic immunosuppressants, his corneal condition improved.

## CASE REPORT

A 71-year-old Chinese male was referred to our department from hematology department of our hospital due to severe dry eyes on August, 2001. He complained dryness, irritating pain and blurred vision of his both eyes in the past few months. Tracing his medical history, he was diagnosed with MM (IgA, IgG, kappa chain) in 1994. Since then, INFα-2b (3-million units, intramuscularly, three times a week) was prescribed until the present. Serological tests showed no evidence of hepatitis B and C infection.

At his first visit, the ocular examinations showed diffuse superficial punctuate epithelial defects (SPK) and filaments in both his corneas. Shirmer test with and without anesthesia showed 0 mm in both eyes. Preservative-free artificial tears (0.3% hyaluronic acid eye drop) were administered every 2 hours regularly but the symptoms of dry eyes persisted. Due to progressive blurred vision, he underwent cataract surgeries of his both eyes in early 2002. Visual acuity improved after surgeries, 20/30 in the right eye and 20/40 in the left, but the SPK became worse.

**Figure 1:**
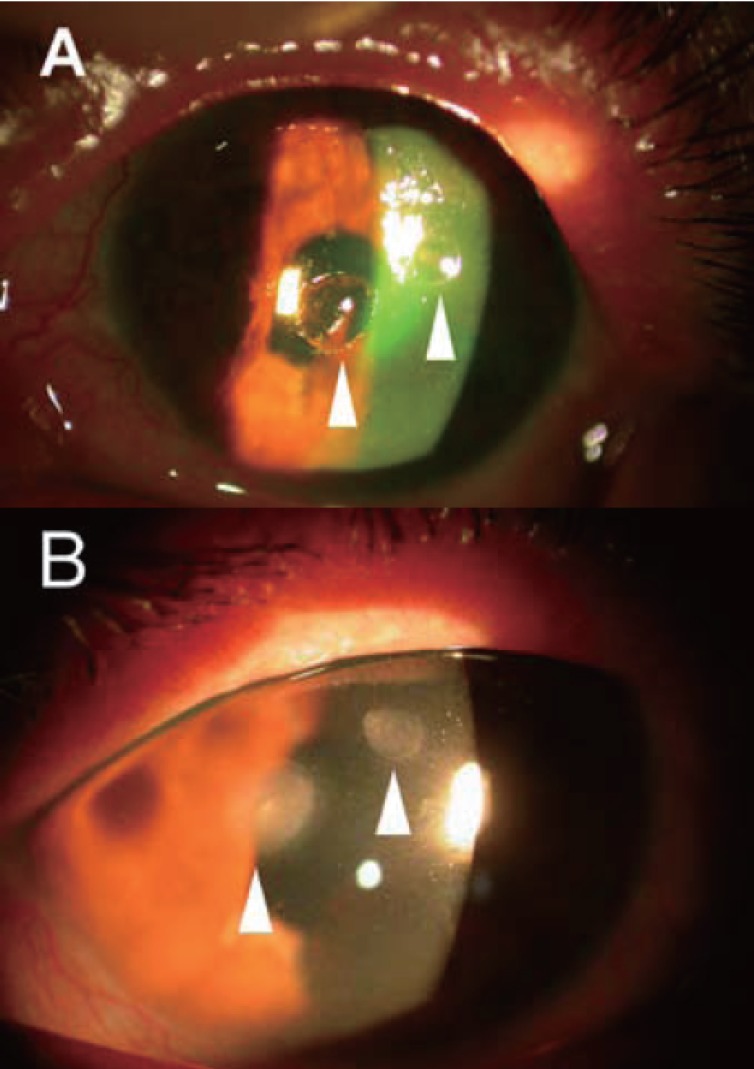
Slit lamp microscope photograph showing (A) sterile melting ulcers (arrowhead) of left cornea with fluorescence staining; (B) healed ulcers with faint scars (arrowhead) after the treatment.

On May, 2004, his oral infection of candidiasis associated with severe dry mouth was found and SS was diagnosed after the salivary gland biopsy was done. He underwent bilateral permanent punctal occlusion (upper and lower punctum) and was advised to wear goggles but in vain. Additional topical immunosuppressants, preservative-free cyclosporine and corticosteroids eye drops were prescribed, but the corneal condition exacerbated. At the visit of June, 2004, he complained severe stinging pain and blurred vision in both eyes. Severe diffuse SPK in both corneas and multiple melting sterile ulcers in his left central cornea were noted (Figure 1A). The vision decreased to 20/200 in the left eye and 20/80 in the right eye. He was hospitalized and treated immediately with additional systemic immunosuppressants, methotrexate 20 mg three times a week and corticosteroid 60 mg per day, in addition to topical lubricant and immunosupressants. Culture report of conjunctival swab corneal scrapings showed no infection. After 2 weeks of admission, the corneal ulcers in the left eye subsided partially and during the two months of follow-up, his corneal ulcers healed completely with residual faint scars (Figure 1B). The vision recovered to 20/50 in the left eye and 20/30 in the right. Systemic immunosuppressants were stopped but the treatment of topical cyclosporine, corticosteroids and lubricants were maintained. He was followed every week in our out patient clinic afterwards with a stable corneal station.

Almost 1 year later, at his visit on April 2005, the corneal condition became worse suddenly. Severe SPK in both corneas and multiple sterile ulcers in his left cornea were noted again. The vision decreased to 20/200 in the left eye and 20/70 in the right. Bilateral lateral permanent tarsorraphy was performed immediately but in vain. Thus, again, the systemic immunosuppressant, methotrexate and corticosteroids, were prescribed. The symptoms improved very quickly this time and ulcers subsided within 2 weeks. Half dosage of systemic immunosuppressant was used as maintenance therapy till the present (longer than 6 months). During this time period, his corneas had maintained stable and the vision was 20/50 in the left eye and 20/30 in the right.

## DISCUSSIONS

INFα-2b is known as an effective immune modulator and has been used for treating HC and MM. Ocular complications with IFN administration include retinal hemorrhage, cotton wool spots, cystoid macular edema, ischemic optic neuropathy, subconjunctival hemorrhage, dry eye, and SS (5). But, according to the published documents, it is still controversial that INFα-2b improves or impairs the tear functions. Most data of the adverse effects of INFα-2b treatment such as dry eyes and dry mouth were from the studies on HC patients (3, 4) and were considered dosage-related (6). However, HC virus could be responsible for a broad range of autoimmune manifestations, including SS and hypothesized a possible pathogenic agent causing lacrimal dysfunction (1, 3). Thus, it can not be ruled out a risk factor for dry eyes.

On the contrary, INFα-2b has been tried to treat SS and shown effective in improving the salivary gland secretion and dry eye symptoms (1, 2). Interestingly, compared with the dosage and duration of INFα-2b administration in HC and MM patients, INFα-2b treatments for SS in these studies were obviously shorter and the dosages were lower (1, 2). Since the side effects of INFα-2b of different regimens have never been compared, it is worthy to evaluate them by a large series study.

According to the literatures, the cases of MM combined with SS are extremely rare (7). Furthermore, patients receiving IFN possibly experience exacerbations of preexisting immune diseases or develop new autoimmune diseases such as systemic lupus erythematosus, rheumatoid arthritis or SS (8). A case of HC developed SS after INFα-2b treatment for 3 months (9). The present case suffered from severe SS after INFα-2b treatment for 10 years and it suggests a possibility of INF-related SS. Under aggressive treatments including topical and systemic immunosuppressants, his corneal condition was controlled. It possibly indicated that his severe dry eyes and corneal melting ulcers might be due to a immune deviation by an unknown autoimmune mechanism related to long-term INFα-2b administration. Besides topical immunosuppressants, systemic immunosuppressant could play a role in treating such corneal complications.
